# Impact of cell lysis treatment before saliva metagenomic DNA extraction on the oral microbiome and the associated resistome

**DOI:** 10.1002/cre2.905

**Published:** 2024-06-28

**Authors:** Supathep Tansirichaiya, Kittikun Songsomboon, Nichamon Chaianant, Wasawat Lertsivawinyu, Mohammed Al‐Haroni

**Affiliations:** ^1^ Department of Microbiology, Faculty of Medicine Siriraj Hospital Mahidol University Bangkok Thailand; ^2^ Department of Clinical Dentistry, Faculty of Health Sciences UiT the Arctic University of Norway Tromsø Norway; ^3^ Centre for New Antimicrobial Strategies UiT the Arctic University of Norway Tromsø Norway; ^4^ School of Life and Environmental Sciences The University of Sydney Sydney Australia; ^5^ Faculty of Dentistry and Research Unit in Mineralized Tissue Reconstruction Thammasat University Pathumthani Thailand

**Keywords:** cell lysis treatment, oral microbiome, oral resistome, saliva metagenomic DNA

## Abstract

**Objectives:**

The human oral microbiome, a complex ecosystem linked to oral and systemic health, harbors a diverse array of microbial populations, including antimicrobial resistance genes (ARGs). As a critical component of the One Health approach to tackle antibiotic resistance, comprehending the oral resistome's composition and diversity is imperative. The objective of this study was to investigate the impact of chemical cell lysis treatment using MetaPolyzyme on the detectability of the oral microbiome, resistome, and DNA quality and quantity.

**Materials and Methods:**

Saliva samples were collected from five healthy individuals, and each of the samples was subjected to DNA extraction with and without the treatment with MetaPolyzyme. Through metagenomic sequencing, we analyzed, assessed, and compared the microbial composition, resistome, and DNA characteristics between both groups of extracted DNA.

**Results:**

Our study revealed that MetaPolyzyme treatment led to significant shifts in the detectability of microbial composition, favoring Gram‐positive bacteria, notably Streptococcus, over Gram‐negative counterparts. Moreover, the MetaPolyzyme treatment also resulted in a distinct change in ARG distribution. This shift was characterized by an elevated proportion of ARGs linked to fluoroquinolones and efflux pumps, coupled with a reduction in the prevalence of tetracycline and β‐lactam resistance genes when compared with the nontreated group. Alpha diversity analysis demonstrated altered species and ARG distribution without affecting overall diversity, while beta diversity analysis confirmed significant differences in the taxonomical composition and oral resistome between treated and nontreated groups.

**Conclusions:**

These findings underscore the critical role of cell lysis treatment in optimizing oral metagenomic studies and enhance our understanding of the oral resistome's dynamics in the context of antimicrobial resistance.

## 1 INTRODUCTION

The human oral microbiome is a complex ecosystem composed of diverse microbial populations that are associated with a range of oral and systemic diseases. Among microbial ecosystems in the human body, the oral cavity ranks as the second most complex, with the identification of over 700 bacterial species (Wade, 2011). Additionally, it has been established as a reservoir for diverse antimicrobial resistance genes (ARGs) and mobile genetic elements (MGEs) (Brooks et al., 2022; Haque et al., 2019; Tansirichaiya et al., 2019). Notably, the oral environment, particularly oral biofilms, serves as an ideal setting for horizontal gene transfer (HGT) and the dissemination of ARGs (Roberts & Kreth, 2014; Roberts & Mullany, 2010). For instance, recent functional metagenomic studies have uncovered *tet*AB(60) in the human oral cavity, conferring resistance to tigecycline, an antibiotic considered a last resort (Reynolds et al., 2016). Therefore, a comprehensive understanding of the oral resistome is imperative for anticipating the potential emergence of resistance and customizing antibiotic therapies.

As only two‐thirds of the oral bacteria can be cultured in the laboratory (Vartoukian et al., 2016), the introduction of culture‐independent methods significantly increases our knowledge of the microbial diversity within the human oral cavity. High‐throughput metagenomic sequencing has revolutionized our ability to study the composition and functional capacity of the oral microbiome and its potential contributions to health and disease (Dewhirst, 2016; Peng et al., 2022; Sedghi et al., 2021). As the oral resistome can easily spread to other parts of the body, compounding the global antimicrobial crisis, it must therefore should be closely studied and monitored to mitigate this threat as well, as it is crucial within the framework of the One Health approach, which recognized the interconnectedness of human health, animal health, and the environment in the spread of antibiotic resistance (Despotovic et al., 2023).

The accurate and reliable extraction of high‐quality DNA from oral samples remains a challenging step in metagenomic analysis, particularly due to the presence of cell walls and extracellular matrices that can impede DNA recovery. Previous study showed similar quality, quantity, and 16s rRNA bacterial profiles of the saliva DNA extracted by using a paramagnetic bead‐based, phenol‐chloroform, and silica column‐based DNA extraction methods (Lim et al., 2017). However, a study to determine the effectiveness of DNA extraction for shotgun metagenomic studies in the oral cavity, especially for the determination of the oral resistome, is still unclear. One promising approach to improve DNA extraction efficiency and yield is the use of chemical cell lysis treatments before DNA extraction like MetaPolyzyme (Tighe et al., 2017), which contains a mixture of 6 enzymes that is intended for the isolation of total DNA for metagenomics studies. While MetaPolyzyme treatments have been used in several metagenomic studies, their effects on the oral microbiome and resistome, that is, the collection of antibiotic resistance genes in a given microbial population, have not yet been fully understood.

While it is well established that different DNA extraction methods can significantly impact the quality and purity of extracted DNA (Lazarevic et al., 2013; Sohrabi et al., 2016; Teng et al., 2018), the specific effects of MetaPolyzyme cell lysis treatments on the oral microbiome and resistome remain less explored. Additionally, cutting‐edge technology proved that different DNA extraction methods yielded different microbial profiles (Ducarmon et al., 2020; Gand et al., 2023; Sui et al., 2020). We thus hypothesized that the cell lysis treatments would improve DNA extraction efficiency and lead to a more comprehensive and accurate analysis of the oral microbiome and resistome. To test this hypothesis, we performed metagenomic sequencing on DNA samples extracted from saliva samples before and after cell lysis treatment and compared the results with those obtained from untreated samples. Our study uniquely focuses on the comprehensive evaluation of chemical cell lysis treatments to understand their impact on the composition and diversity of the oral microbiome and resistome.

Our findings have important implications for the development of optimized protocols for oral metagenomic analysis and shed light on the potential benefits and limitations of chemical cell lysis treatments for this purpose.

## 2 MATERIALS AND METHODS

### 2.1 Saliva sample collection

Saliva samples were collected from five healthy volunteers who visited the dental clinic at the Thammasat University Hospital, Thailand. The volunteers were subjected to oral evaluation and had not received antibiotic treatment for at least 3 months and abstained from drinking, eating, and brushing at least 1 h before the collection. Ethical approval was obtained from the Ethical Review Sub‐Committee Board of Human Research Involving Science, Thammasat University, Thailand (COA No. 163/2562). Written consent was obtained from volunteers after properly informed about the study. Saliva secretion was stimulated by using paraffin gum, and at least, 2 mL of saliva was collected into Norgen's Saliva DNA Collection, Preservation and Isolation Kit (Norgen Biotek Corp), which can preserve and maintain DNA at ambient temperature for at least a year. The samples were anonymized and stored at room temperature until processing.

### 2.2 Saliva metagenomic DNA extraction

Saliva samples were mixed by inverting the tubes several times and then aliquoting with 550 µL total volumes of a saliva sample into two 2‐mL microcentrifuge tubes. Fifty microliters of MetaPolyzyme (Merck) were added to the first tube, while 50 µL phosphate‐buffered saline (PBS) was added to another tube. All tubes were incubated at 35°C for 5 h. The DNA samples were then extracted with QIAcube (Qiagen), using a modified protocol from QIAamp® DNA Mini QIAcube Kit which increased the volume of proteinase K, Buffer AL, and ethanol to 40, 400, and 400 µL, respectively, as the starting volume was increased from the original protocol. The integrity, purity, and concentration of the extracted DNA samples were determined by agarose gel electrophoresis, a NanoDrop spectrophotometer (Thermo Fisher Scientific), and a Qubit Fluorometer (Thermo Fisher Scientific), respectively.

### 2.3 Metagenomic sequencing and data processing

The library construction and metagenomic sequencing of the extracted DNA products were performed at Beijing Genomics Institute (Shenzhen, China) by using the DNBSEQ platform (150‐bp pair‐end reads) to generate at least 20 GB of data per sample. Human DNA was filtered out by using Fastq screen version 0.14.1 (Wingett & Andrews, 2018) to match the reads with the human reference genome GRCh38 (August 2020). The reads were repaired by using Bbmap version 38.84 (Bushnell et al., 2017) to remove singleton reads that their pair were discarded by the Fastq screen. Adapter contamination and low‐quality reads were removed by using AfterQC version 0.9.7 with default parameters (Chen et al., 2017).

### 2.4 Microbiome and resistome analyses

Microbiome analysis was performed by using KrakenUniq taxonomic classification tools (version 0.5.7) (Breitwieser et al., 2018). Reads processed by AfterQC were classified using the Kraken database built from complete genomes in RefSeq from January 2020 for bacteria and archaea. The accuracy of taxonomical classification was enhanced by using krakenuniq‐filter script with a threshold of 0.05. KrakenUniq's outputs were generated by using krakenuniq‐report script. Resistome analysis was performed on the quality‐filtered reads by using the AMRPlusPlus resistome analysis pipeline tools version 2 (Doster et al., 2019). ARGs requiring single‐nucleotide polymorphism (SNP) confirmation were filtered out from the reports, and the gene coverage/fraction threshold was set at 80%. The AMR results were normalized by using the following equation (Li et al., 2015):

$$\text{Abundance}\;=\sum_{1}^{n}\frac{N_{\text{ARG}-\text{likesequence}}\times L_{\text{reads}}/L_{\text{ARGreferencesequence}}}{N_{16\text{Ssequence}}\times L_{\text{reads}}/L_{16\text{Ssequence}}},$$

where *N*_ARG‐likesequence_ and *N*_16Ssequence_ are the number of the ARG‐like sequence annotated by AMRPlusplus and the number of the 16S sequence identified by Metaxa version 2.2 (Bengtsson et al., 2011), respectively, while *L*_reads_, *L*_ARGreferencesequence_, and *L*_16Ssequence_ are the length of sequencing reads, the sequence length of the corresponding specific ARG reference sequence in MEGARes databases, and the average length of the 16S sequence, respectively.

### 2.5 Alpha and beta diversity and statistical test

Both AMR and Kraken counts were normalized based on shifts in the distributions of counts using normalization of Cumulative Sum Scaling (CSS) in R (version 4.2.1) via the metagenomeSeq package (version 4.1‐8) (Paulson et al., 2013). For alpha diversity, the normalized abundances from AMRPlusplus and Kraken in each level of classification were used to calculate Shannon and Simpson indices along with evenness in R using the Phyloseq package (version 1.9.2) (McMurdie & Holmes, 2013). Beta diversity was analyzed using nonmetric multidimensional scaling (NMDS) analysis based on Bray–Curtis dissimilarity in R via Vegan (version 2.6‐4) (Dixon, 2003). The separation between treatment groups was tested with PerMANOVA via Vegan. All of the custom R scripts for analysis and graphic generation were available on GitHub via https://github.com/skittikun/Saliva_Metagenomic_DNA.git.

## 3 RESULTS

### 3.1 General information

**Figure 1:**
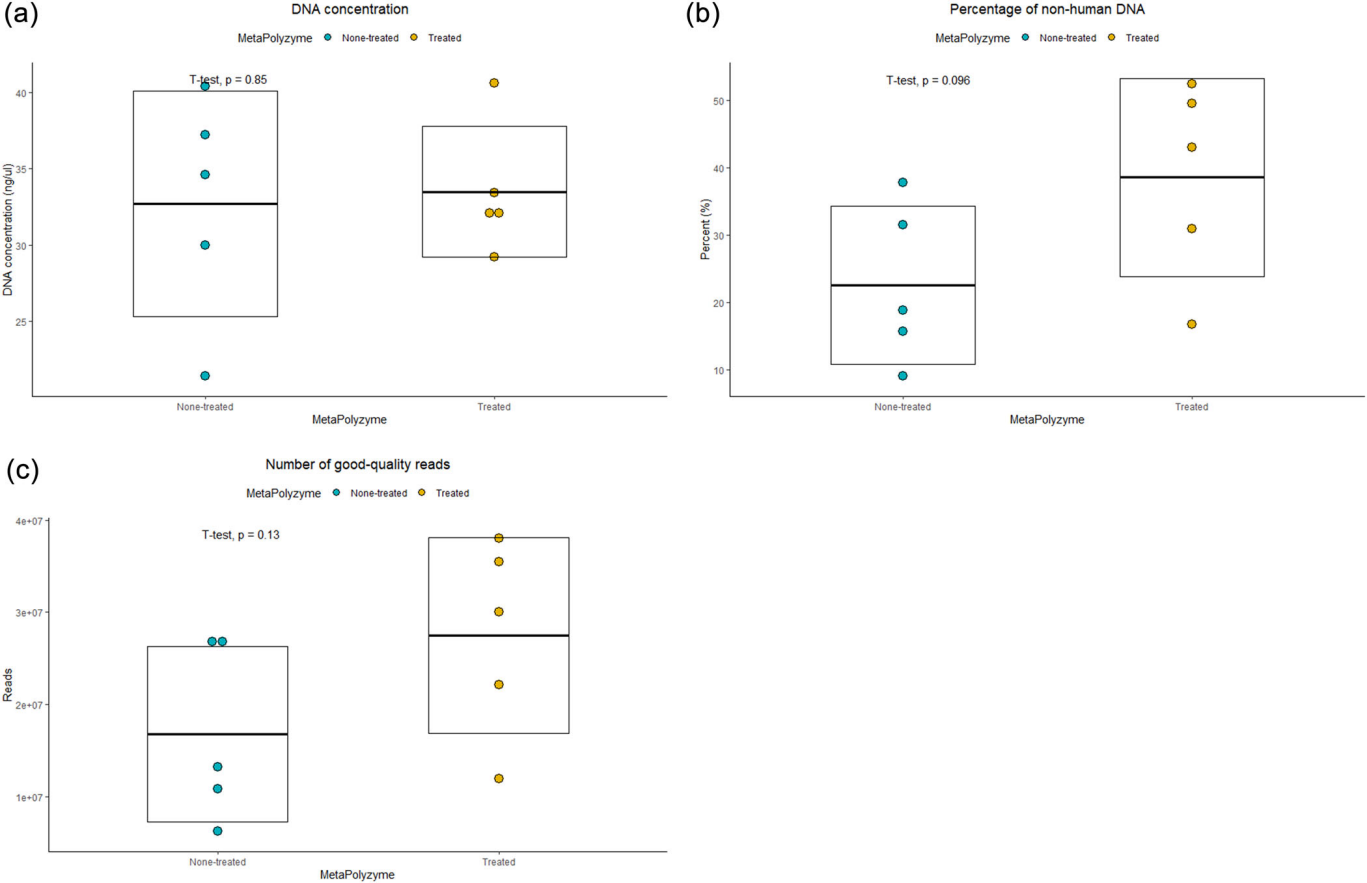
Comparison of general characteristics in extracted saliva metagenomes between enzyme‐treated and nontreated groups. Dot plots illustrate (a) DNA concentration, (b) the percentage of nonhuman DNA, and (c) the number of high‐quality reads in both nontreated (Blue) and enzyme‐treated groups (Yellow). The middle line within each box represents the mean values, while the upper and lower lines denote the mean values with their respective standard deviations.

Five saliva samples were subjected to two extraction conditions: with and without MetaPolyzyme treatment, resulting in a total of 10 extracted DNA samples for sequencing. The average DNA concentration across all samples was 33.1 ng/μL, ranging from 21.4 to 40.6 ng/μL, with similar concentrations observed in both the enzyme‐treated group (average of 33.5 ng/μL) and the nontreated group (average of 32.7 ng/μL) (Figure 1a and Table 1).

**Table 1:** Statistics of the extracted metagenomic DNA and sequencing data.

Sample ID	MetaPolyzyme	DNA concentration (ng/μL)	Number of reads (reads)			Bioinformatics hits (hits)^a^		
			Raw sequence reads	Nonhuman DNA	Reads passed QC	Phylum hits	AMR hits	16s rRNA hits
8019	Treated	33.4	145,498,024	76,175,152	76,036,166	24,786,728	71,402	59,410
	Nontreated	34.6	140,302,012	52,779,030	52,681,078	16,056,284	35,892	33,238
8035	Treated	32.4	143,766,854	44,306,390	44,219,264	15,958,973	50,799	33,621
	Nontreated	37.2	140,301,722	21,856,684	21,808,206	7,250,405	17,462	14,377
8053	Treated	29.2	140,303,438	60,249,462	60,111,518	23,027,393	88,765	52,509
	Nontreated	21.4	142,034,834	26,617,996	26,573,646	9,703,433	21,928	19,177
8075	Treated	40.6	143,766,798	71,027,646	70,867,208	22,340,731	90,705	54,640
	Nontreated	30.0	174,230,434	54,721,854	54,542,900	16,403,991	63,780	33,086
8077	Treated	31.8	143,765,750	23,972,680	23,915,868	9,025,354	34,675	19,798
	Nontreated	40.4	140,303,030	12,538,640	12,512,462	3,978,411	9242	8283

^a^ Phylum, AMR, and 16s rRNA hits were analyzed by using KrakenUniq, AMRPlusPlus, and Metaxa, respectively.

A total of 727 million paired‐end DNA sequences were generated, with an average of 72.7 million sequences (range: 70.2–87.1 million). After filtering human DNA from the raw reads, an average of 30.5% nonhuman DNA was observed, with a range from 9.0% to 52.5%. Notably, the nontreated group exhibited lower levels of nonhuman DNA, averaging 22.5%, compared with the enzyme‐treated group with an average of 38.5% (Figure 1b and Supporting Information: Table S1). Subsequent quality control processes with AfterQC retained a total of 221 million paired‐end reads, with an average of 22.2 million reads (range: 6.3–38.0 million). The enzyme‐treated group had an average of 27.5 million reads, while the nontreated group averaged 16.8 million reads (Figure 1c).

### 3.2 Effects of chemical cell lysis treatment on microbiome composition

**Figure 2:**
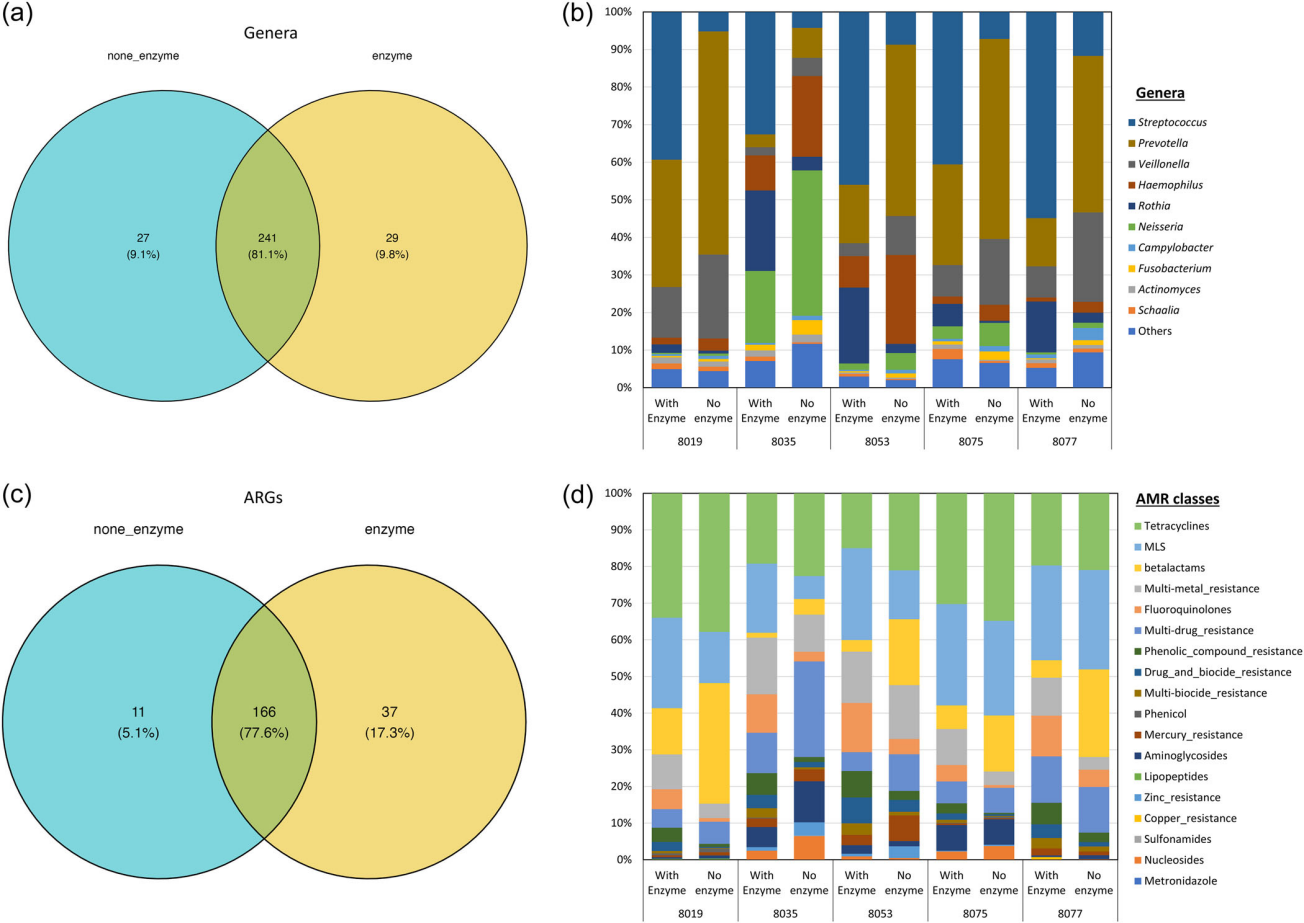
Taxonomical and resistance profile comparison between enzyme‐treated and nontreated groups. Venn diagrams illustrating the identified (a) genera and (c) ARGs in nontreated (blue) and enzyme‐treated groups (yellow). Stacked bar charts display the relative abundance of the taxonomical composition of (b) the top 10 genera and (d) AMR drug classes.

Taxonomic assignments were performed with KrakenUniq on the preprocessed sequencing data, identifying bacterial and archaeal reads in the saliva metagenome at an average of 72.6% (range: 65.4%–81.6%). Taxonomic classification at the genus level assigned the reads to a total of 297 genera, of which 241 genera (81.1%) were shared between the samples treated and not treated with MetaPolyzyme, and 29 (9.8%) and 27 (9.1%) genera were unique to each group, respectively (Figure 2a and Supporting Information: Table S2).

**Figure 3:**
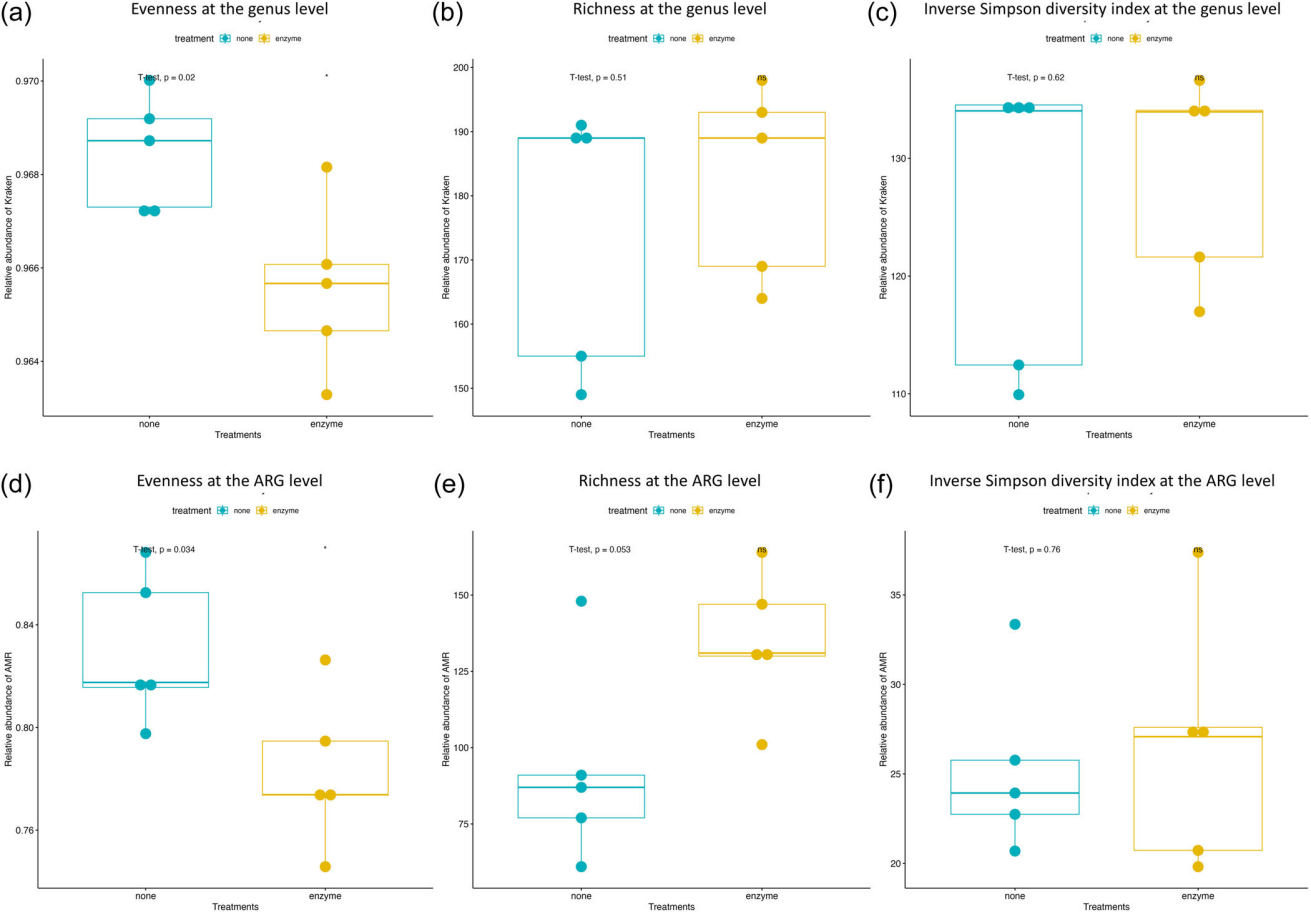
Alpha diversity analysis of the oral microbiome and resistome. Box plots comparing alpha diversity indices at the genus level (a–c) and ARG level (d–f) between the nontreated group (blue) and enzyme‐treated group (yellow). The alpha diversity indices assessed include evenness (a and d), richness (b and e), and the inverse Simpson diversity index (c and f).

The enzyme‐treated samples predominantly contained *Streptococcus*, Gram‐positive bacilli bacteria, while nontreated samples predominantly contained *Prevotella*, *Neisseria*, and *Veillonella*, all of which are Gram‐negative bacteria (Figure 2b). Inverse Simpson index, Shannon diversity index, evenness, and richness were calculated at each taxonomical level and are summarized in Supporting Information: Table S3. Evenness between the MetaPolyzyme‐treated group and the nontreated group was statistically different at all taxonomical levels, except at the phylum level, while the inverse Simpson index, Shannon diversity index, and richness were not statistically different among both groups at all levels (Figure 3a–c and Supporting Information: Table S3).

**Figure 4:**
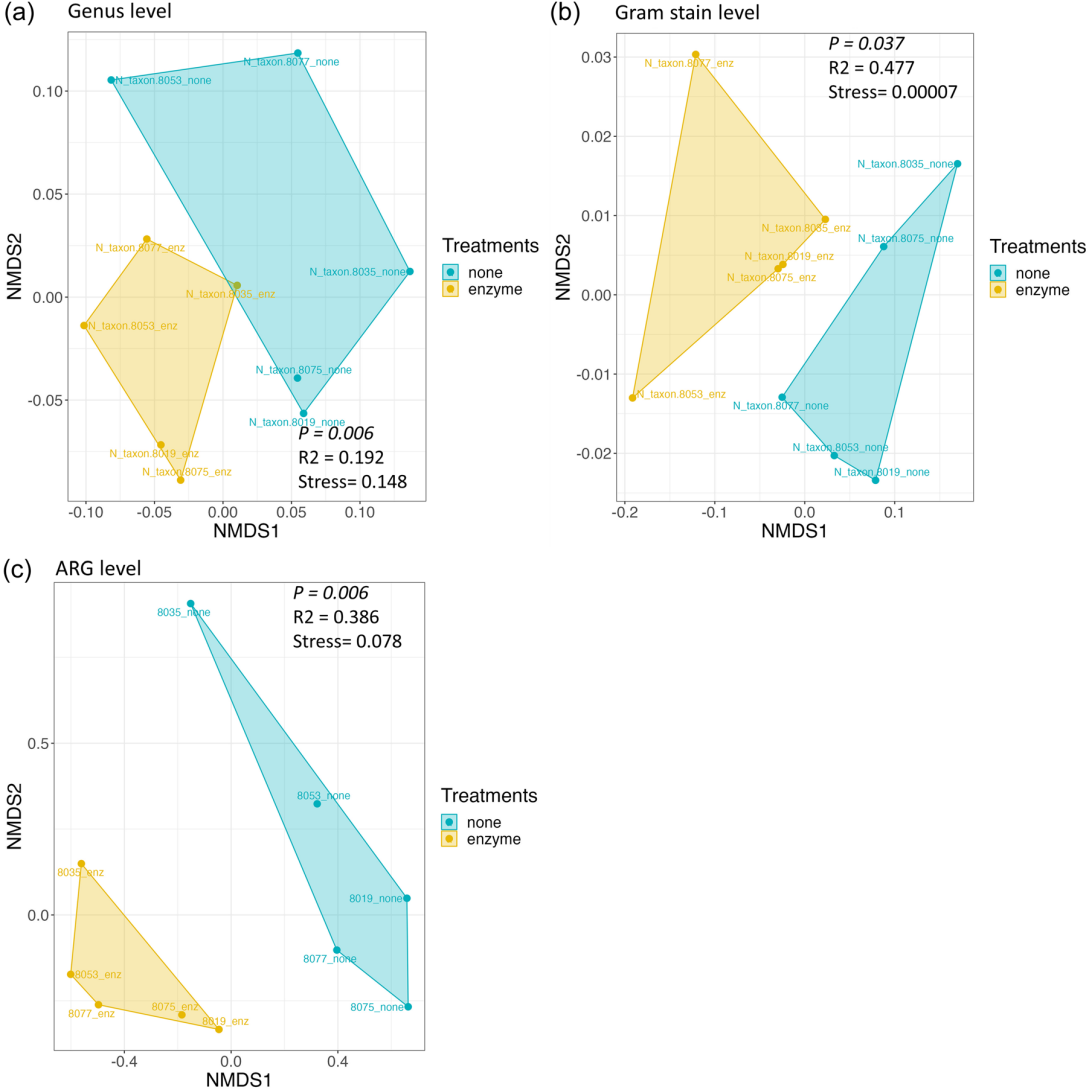
Beta diversity of the oral microbiome and resistome. Bray–Curtis dissimilarity comparisons between the nontreated group (blue) and enzyme‐treated group (yellow) are presented at the (a) genus level, (b) Gram stain level, and (c) ARG level using NDMS.

Bray–Curtis dissimilarity was performed at the genus level to determine the dissimilarity among the treated and nontreated samples, as shown in Figure 4a. NMDS analysis showed that the saliva microbiome between both groups was significantly different at the phylum, class, and genus levels, with the highest significance at the genus level (*p* = .006) (Supporting Information: Table S4). Since the relative abundance results suggested that both groups also exhibited different microbiome compositions of Gram‐positive and Gram‐negative bacteria, beta diversity was also assessed at this level, confirming the differences between both groups (*p* = .037) (Figure 4b).

### 3.3 Effects of chemical cell lysis treatment on ARG composition

The ARG‐associated reads were identified and matched with the MEGARes AMR database using AMRplusplus version 2.0, with a gene fraction threshold set at 80%. The results showed that, from all samples, 214 ARGs were identified, where 166 ARGs (77.6%) were shared between both groups (Figure 2c,d and Supporting Information: Table S5), spanning across 10 AMR classes, including tetracyclines, MLS, β‐lactams, multimetal resistance efflux pumps, multidrug resistance efflux pumps, multibiocide efflux pumps, drug and biocide efflux pumps, phenolic compounds, fluoroquinolones, mercury, and aminoglycosides. The number of unique ARGs in enzyme‐treated and none‐treated groups was 37 (17.3%) and 11 (5.1%), respectively (Figure 2c).

Comparing the resistome composition between the two groups revealed that all samples in the treated group contained a higher proportion of ARGs for fluoroquinolones, phenolic compounds, multi‐biocide efflux pumps, and drug and biocide efflux pumps. They also had a lower proportion of ARGs against tetracyclines and β‐lactams compared with their corresponding samples in the nontreated groups (Figure 2d). Inverse Simpson index, Shannon diversity index, evenness, and richness were also calculated at the ARG level and summarized in Supporting Information: Table S3, which showed that the evenness index between both groups was statistically different at the ARG level (*p* = .034), while the others were not statistically different (Figure 3d–f). The dissimilarity between the treated and nontreated groups at the ARG level was determined using Bray–Curtis dissimilarity, which showed that the saliva microbiome between both groups was significantly different (*p* = .006), as shown in Figure 4c.

## 4 DISCUSSION

The oral microbiome is a complex and diverse ecosystem that plays a crucial role in maintaining oral health and potentially influencing systemic health, which also presents as an important reservoir for ARGs. In this study, we aimed to investigate the effects of cell lysis treatment on the composition and diversity of the oral microbiome and resistome, as well as the quality and quantity of extracted DNA. Recognizing the contributions of previous studies that have explored the effects of various treatments on DNA extraction from the salivary microbiome and other microbiomes (Ducarmon et al., 2020; Gand et al., 2023; Lazarevic et al., 2013; Sohrabi et al., 2016; Sui et al., 2020; Teng et al., 2018), we hypothesized that the MetaPolyzyme treatment would enhance the efficiency of DNA extraction, thereby potentially providing a more representative analysis of the oral microbiome and resistome.

Clinical sample collection isolates microbes from the external environment, resulting in a fixed microbial composition at the time of acquisition, and remains stable unless post‐collection contamination or growth occurs. DNA extraction plays a crucial role in shaping the results of metagenomic studies on the oral microbiome, but it's important to note that different extraction methods primarily affect DNA availability and not the actual composition of the microbial community. Consequently, observed levels of DNA from different oral microbiome members are approximations, highlighting the limitations of accurately capturing the true composition.

A silica column‐based extraction method, like in this study, utilizes a lysis buffer and proteinase K, which are designed to break down bacterial cell membranes and disrupt interfering proteins, enabling the lysis of bacterial cells and the release of their DNA (Salazar & Asenjo, 2007). However, it is important to acknowledge that certain bacteria, such as those with thick peptidoglycan cell walls like Gram‐positive bacteria, may be less efficiently extracted using this method and could potentially be underrepresented in the subsequent metagenomic analysis (Kauffmann et al., 2004). To enhance inclusiveness, additional mechanical or enzymatic methods may be necessary before extraction. This is especially important when investigating the oral resistome to obtain an accurate representation of resistance genes in the human oral cavity. Mechanical methods like bead beating or sonication can exert physical force on DNA molecules, which may result in shearing if the conditions are not carefully controlled (Burden, 2012). Therefore, if preserving DNA integrity is crucial, as is the case for techniques like long‐read metagenomic sequencing, enzymatic methods may be preferred.

In our study, we determined the effects of the enzymatic method using MetaPolyzyme, which comprises a mixture of microbial lytic enzymes, including achromopeptidase, chitinase, lyticase, lysostaphin, lysozyme, and mutanolysin (Tighe et al., 2017). It was designed to facilitate the breakdown of cell wall components, leading to more efficient lysis of resulting spheroplasts or protoplasts, which have been used in several human‐related metagenomic studies such as in the gut, bronchoalveolar lavage fluid, and oral cavity (Al‐Hebshi et al., 2019; Baraniya et al., 2020; Maghini et al., 2021; Saladié et al., 2020). This approach has been previously tested with metagenomic nanopore sequencing in human stool samples and has also been employed for the rapid detection of pathogens and ARGs in urine samples through nanopore sequencing (Moss et al., 2020; Zhang, Huang, et al., 2022). Building upon this foundation, our study aims to extend the knowledge base by specifically investigating the impact of MetaPolyzyme on the oral resistome, an area that has not yet been exclusively examined.

Our results indicated that cell lysis treatment significantly influenced the detectability and representation of microbial DNA, thereby affecting the observed composition and diversity in the analysis of the oral microbiome. Taxonomic analysis at the genus level indicated that the treatment resulted in an apparent shift in the predominant bacterial genera, from a predominance of Gram‐negative bacteria in the nontreated group to Gram‐positive bacilli bacteria in the enzyme‐treated samples. These results were in accordance with previous studies that reported the presence of *Streptococcus* as one of the dominant oral bacteria, where 18 species reside within the oral cavity, such as *S. mutans*, *S. oralis*, and *S. sanguinis (*Baty et al., 2022; Okahashi et al., 2022).

Aligning with previous studies demonstrating MetaPolyzyme treatment's ability to improve the recovery of DNA from Gram‐positive bacteria (Tighe et al., 2017; Zhang, Chen, et al., 2020; Zhang, Huang, et al., 2022), our findings suggest that the enzyme treatment may enhance the recovery of DNA from Gram‐positive bacteria, leading to an apparent increase in their representation relative to Gram‐negative bacteria in the analyzed oral microbiome samples. Additionally, the sequencing results also showed that a lower proportion of human DNA to nonhuman DNA was observed when saliva samples were treated with the enzyme, which could be associated with the increased release of bacterial DNA in the extraction process as well.

Alpha diversity analysis on the taxonomical classification suggests that enzymatic treatment with MetaPolyzyme did not have a substantial impact on species diversity or the number of unique species within each sample but rather influenced the relative abundance of each species in the samples. This effect could be attributed to the enzyme treatment's ability to enhance cell lysis, thereby affecting the even distribution of species in the samples. Beta diversity analysis using Bray–Curtis dissimilarity further highlighted differences between the treated and nontreated groups, where the saliva microbiome between both groups was significantly different at the phylum, class, and genus levels, with the highest significance observed at the genus level. This observation highlights the significant impact of cell lysis treatment on the detectability and representation of microbial DNA within the oral metagenome.

The effects of cell lysis treatment on ARG composition in the oral metagenome were also examined in this study, spanning over 10 AMR classes, indicating the diversity of resistance mechanisms present in the oral cavity, similar to the reports from other studies (Anderson et al., 2023; Caselli et al., 2020; Sukumar et al., 2023; Tansirichaiya et al., 2016, 2017; Tansirichaiya et al., 2022; Wigand et al., 2021). The alpha diversity indices at the ARG level also revealed similar results as in the microbiome analysis, where the evenness was statistically and significantly different between the treated and nontreated groups, but there were no significant differences in the inverse Simpson index and richness. This indicates that the treatment influenced the even distribution of ARGs without significantly affecting their overall diversity. Bray–Curtis dissimilarity at the ARG level further supported the notion that cell lysis treatment altered the resistome composition. The dissimilarity between the treated and nontreated groups at the ARG level was significant, emphasizing the role of treatment in shaping the oral resistome.

These findings collectively underscore the importance of considering cell lysis treatment in studies of the oral microbiome and resistome. The observed differences in the representation of microbial taxa and resistance gene distribution underscore the importance of sample processing techniques in influencing the results of metagenomic analyses, reflecting variations in DNA extraction efficiency. It was shown previously that different extraction methods could have a great impact and influence the detected microbiome structure generated from the samples (Costea et al., 2017; Stinson et al., 2018). It was also suggested to be dependent on the characteristics of the samples, as they are all different, such as in the types of microbes and the nature of the samples. For instance, when investigating soil and fecal microbiomes, additional decontamination steps are often necessary to remove inorganic and organic particles that might impede the effectiveness of cell lysis and potentially disrupt subsequent DNA purification and enzymatic processes (Davis et al., 2019; Ducarmon et al., 2020; Gand et al., 2023; Lim et al., 2020; Sui et al., 2020). In contrast, DNA extraction from aquatic environmental samples, which typically contain significantly lower levels of such interfering substances, can be carried out directly using protocols designed for pure culture (v. Wintzingerode et al., 1997). It is, therefore, crucial to test and optimize for the most suitable extraction approaches to obtain comprehensive and accurate data for downstream analysis.

Typically, investigating the microbiome by targeted metagenomics through 16S rRNA sequencing which uses DNA primers amplifying tarting region of 16S rRNA such as V3‐V4 variable regions for gut microbiome and V1‐V2 for oral microbiome (Na et al., 2023). Therefore, the contamination of host DNA in the extracted DNA would not affect the sequencing depth and downstream analysis much. However, to investigate the resistome, the extracted DNA has to be sequenced through shotgun metagenomics to analyze all DNA fragments without amplification to comprehensively analyze ARGs.

In shotgun metagenomics, nonbacterial DNA, such as host DNA, can consume sequencing reads and diminish the sequencing depth of bacterial DNA (Greathouse et al., 2019). This is particularly relevant in the human oral cavity, where contamination with human cells is common. Given that the human genome is roughly a thousand times larger than the average bacterial genome, it can easily overshadow microbial sequencing reads. To address this issue, two primary approaches can be employed: generating more sequencing reads to achieve sufficient sequencing depth for bacterial DNA analysis or depleting host DNA during an additional extraction step (Marotz et al., 2018), which both can be costly. In our study, we demonstrated a reduction in the proportion of human DNA in saliva samples treated with MetaPolyzyme compared with the untreated group. This is likely attributed to the enzyme's ability to enhance the lysis of bacterial cells, resulting in an increased yield of bacterial DNA within the samples.

While our study offers valuable insights into the impact of MetaPolyzyme treatment on the detectability of the oral microbiome and resistome, it is crucial to acknowledge certain limitations. The study was conducted with a relatively small sample size, comprising saliva samples from five healthy individuals. While this enabled a detailed analysis for comparing the microbiome and resistome between the treated and nontreated groups, the generalizability of the findings to broader populations may be constrained. Future studies with larger sample sizes are warranted to confirm and extend the applicability of our conclusions. Furthermore, by examining diverse populations and accounting for individual variations in the oral microbiome, we can gain a more nuanced understanding of how MetaPolyzyme treatment affects the recovery and representation of microbial DNA in metagenomic analyses.

## 5 CONCLUSIONS

This study provides valuable insights into the effects of cell lysis treatment on the detection and representation of the oral microbiome and resistome. The observed shifts in microbial composition and resistance gene distribution highlight the need for standardized protocols in metagenomic studies and underscore the importance of continued research in understanding the dynamics of the oral resistome in the context of antimicrobial resistance.

## AUTHOR CONTRIBUTIONS

Supathep Tansirichaiya and Mohammed Al‐Haroni were involved in the design of experiments and conceptualizing. Supathep Tansirichaiya, Kittikun Songsomboon, and Wasawat Lertsivawinyu were involved in data analyses, visualization, and interpretation of results. Supathep Tansirichaiya and Nichamon Chaianant were involved in clinical sample collection and processing. Manuscript was drafted by Supathep Tansirichaiya, and all authors contributed toward the editing of the manuscript as well as the approval of the final manuscript.

## CONFLICT OF INTEREST STATEMENT

The authors declare no conflict of interest.

## ETHICS STATEMENT

The collection of saliva samples was approved by the Ethical Review Sub‐Committee Board of Human Research Involving Science, Thammasat University, Thailand (COA No. 163/2562). All samples were anonymized and written consent were obtained before the collection of samples.

## Supporting information

Supporting information.

## Data Availability

The data could be obtained upon request to the corresponding author. The saliva metagenome data of all samples are available at the NCBI under BioProject of PRJNA1018099.

## References

[cre2905-bib-0001] Al‐Hebshi, N. N. , Baraniya, D. , Chen, T. , Hill, J. , Puri, S. , Tellez, M. , Hasan, N. A. , Colwell, R. R. , & Ismail, A. (2019). Metagenome sequencing‐based strain‐level and functional characterization of supragingival microbiome associated with dental caries in children. Journal of Oral Microbiology, 11, 1557986. 30671194 10.1080/20002297.2018.1557986 PMC6327923

[cre2905-bib-0002] Anderson, A. C. , von Ohle, C. , Frese, C. , Boutin, S. , Bridson, C. , Schoilew, K. , Peikert, S. A. , Hellwig, E. , Pelz, K. , Wittmer, A. , Wolff, D. , & Al‐Ahmad, A. (2023). The oral microbiota is a reservoir for antimicrobial resistance: Resistome and phenotypic resistance characteristics of oral biofilm in health, caries, and periodontitis. Annals of Clinical Microbiology and Antimicrobials, 22, 37. 37179329 10.1186/s12941-023-00585-z PMC10183135

[cre2905-bib-0003] Baraniya, D. , Chen, T. , Nahar, A. , Alakwaa, F. , Hill, J. , Tellez, M. , Ismail, A. , Puri, S. , & Al‐Hebshi, N. N. (2020). Supragingival mycobiome and inter‐kingdom interactions in dental caries. Journal of Oral Microbiology, 12, 1729305. 32158514 10.1080/20002297.2020.1729305 PMC7048226

[cre2905-bib-0004] Baty, J. J. , Stoner, S. N. , & Scoffield, J. A. (2022). Oral commensal streptococci: Gatekeepers of the oral cavity. Journal of Bacteriology, 204, e00257–22. 36286512 10.1128/jb.00257-22 PMC9664950

[cre2905-bib-0005] Bengtsson, J. , Eriksson, K. M. , Hartmann, M. , Wang, Z. , Shenoy, B. D. , Grelet, G. A. , Abarenkov, K. , Petri, A. , Alm Rosenblad, M. , & Nilsson, R. H. (2011). Metaxa: A software tool for automated detection and discrimination among ribosomal small subunit (12S/16S/18S) sequences of archaea, bacteria, eukaryotes, mitochondria, and chloroplasts in metagenomes and environmental sequencing datasets. Antonie Van Leeuwenhoek, 100, 471–475. 21674231 10.1007/s10482-011-9598-6

[cre2905-bib-0006] Breitwieser, F. P. , Baker, D. N. , & Salzberg, S. L. (2018). KrakenUniq: Confident and fast metagenomics classification using unique k‐mer counts. Genome Biology, 19, 198. 30445993 10.1186/s13059-018-1568-0 PMC6238331

[cre2905-bib-0007] Brooks, L. , Narvekar, U. , McDonald, A. , & Mullany, P. (2022). Prevalence of antibiotic resistance genes in the oral cavity and mobile genetic elements that disseminate antimicrobial resistance: A systematic review. Molecular Oral Microbiology, 37, 133–153. 35674142 10.1111/omi.12375

[cre2905-bib-0008] Burden, D. W. (2012). Guide to the disruption of biological samples‐2012. Random Primers, 12, 1–25.

[cre2905-bib-0009] Bushnell, B. , Rood, J. , & Singer, E. (2017). BBMerge – Accurate paired shotgun read merging via overlap. PLoS One, 12, e0185056. 29073143 10.1371/journal.pone.0185056 PMC5657622

[cre2905-bib-0010] Caselli, E. , Fabbri, C. , D'Accolti, M. , Soffritti, I. , Bassi, C. , Mazzacane, S. , & Franchi, M. (2020). Defining the oral microbiome by whole‐genome sequencing and resistome analysis: The complexity of the healthy picture. BMC Microbiology, 20, 120. 32423437 10.1186/s12866-020-01801-y PMC7236360

[cre2905-bib-0011] Chen, S. , Huang, T. , Zhou, Y. , Han, Y. , Xu, M. , & Gu, J. (2017). AfterQC: Automatic filtering, trimming, error removing and quality control for fastq data. BMC Bioinformatics, 18, 80. 28361673 10.1186/s12859-017-1469-3 PMC5374548

[cre2905-bib-0012] Costea, P. I. , Zeller, G. , Sunagawa, S. , Pelletier, E. , Alberti, A. , Levenez, F. , Tramontano, M. , Driessen, M. , Hercog, R. , Jung, F. E. , Kultima, J. R. , Hayward, M. R. , Coelho, L. P. , Allen‐Vercoe, E. , Bertrand, L. , Blaut, M. , Brown, J. R. M. , Carton, T. , Cools‐Portier, S. , … Bork, P. (2017). Towards standards for human fecal sample processing in metagenomic studies. Nature Biotechnology, 35, 1069–1076. 10.1038/nbt.3960 28967887

[cre2905-bib-0013] Davis, A. , Kohler, C. , Alsallaq, R. , Hayden, R. , Maron, G. , & Margolis, E. (2019). Improved yield and accuracy for DNA extraction in microbiome studies with variation in microbial biomass. Biotechniques, 66, 285–289. 31124702 10.2144/btn-2019-0016

[cre2905-bib-0014] Despotovic, M. , de Nies, L. , Busi, S. B. , & Wilmes, P. (2023). Reservoirs of antimicrobial resistance in the context of One Health. Current Opinion in Microbiology, 73, 102291. 36913905 10.1016/j.mib.2023.102291 PMC10265130

[cre2905-bib-0015] Dewhirst, F. E. (2016). The oral microbiome: Critical for understanding oral health and disease. Journal of the California Dental Association, 44, 409–410. 27514152 PMC7061343

[cre2905-bib-0016] Dixon, P. (2003). VEGAN, a package of R functions for community ecology. Journal of Vegetation Science, 14, 927–930.

[cre2905-bib-0017] Doster, E. , Lakin, S. M. , Dean, C. J. , Wolfe, C. , Young, J. G. , Boucher, C. , Belk, K. E. , Noyes, N. R. , & Morley, P. S. (2019). MEGARes 2.0: A database for classification of antimicrobial drug, biocide and metal resistance determinants in metagenomic sequence data. Nucleic Acids Research, 48, D561–D569. 10.1093/nar/gkz1010 PMC7145535 31722416

[cre2905-bib-0018] Ducarmon, Q. R. , Hornung, B. V. H. , Geelen, A. R. , Kuijper, E. J. , & Zwittink, R. D. (2020). Toward standards in clinical microbiota studies: Comparison of three DNA extraction methods and two bioinformatic pipelines. mSystems, 5, 10‐1128. 10.1128/mSystems.00547-19 PMC7018525 32047058

[cre2905-bib-0019] Gand, M. , Bloemen, B. , Vanneste, K. , Roosens, N. H. C. , & De Keersmaecker, S. C. J. (2023). Comparison of 6 DNA extraction methods for isolation of high yield of high molecular weight DNA suitable for shotgun metagenomics Nanopore sequencing to detect bacteria. BMC Genomics, 24, 438. 37537550 10.1186/s12864-023-09537-5 PMC10401787

[cre2905-bib-0020] Greathouse, K. L. , Sinha, R. , & Vogtmann, E. (2019). DNA extraction for human microbiome studies: The issue of standardization. Genome Biology, 20, 212. 31639026 10.1186/s13059-019-1843-8 PMC6802309

[cre2905-bib-0021] Haque, M. , Sartelli, M. , & Haque, S. (2019). Dental infection and resistance—Global health consequences. Dentistry Journal. 7, 22. 30823670 10.3390/dj7010022 PMC6473604

[cre2905-bib-0022] Kauffmann, I. M. , Schmitt, J. , & Schmid, R. D. (2004). DNA isolation from soil samples for cloning in different hosts. Applied Microbiology and Biotechnology, 64, 665–670. 14758515 10.1007/s00253-003-1528-8

[cre2905-bib-0023] Lazarevic, V. , Gaïa, N. , Girard, M. , François, P. , & Schrenzel, J. (2013). Comparison of DNA extraction methods in analysis of salivary bacterial communities. PLoS One, 8, e67699. 23844068 10.1371/journal.pone.0067699 PMC3701005

[cre2905-bib-0024] Li, B. , Yang, Y. , Ma, L. , Ju, F. , Guo, F. , Tiedje, J. M. , & Zhang, T. (2015). Metagenomic and network analysis reveal wide distribution and co‐occurrence of environmental antibiotic resistance genes. The ISME Journal, 9, 2490–2502. 25918831 10.1038/ismej.2015.59 PMC4611512

[cre2905-bib-0025] Lim, M. Y. , Park, Y.‐S. , Kim, J.‐H. , & Nam, Y.‐D. (2020). Evaluation of fecal DNA extraction protocols for human gut microbiome studies. BMC Microbiology, 20, 212. 32680572 10.1186/s12866-020-01894-5 PMC7367376

[cre2905-bib-0026] Lim, Y. , Totsika, M. , Morrison, M. , & Punyadeera, C. (2017). The saliva microbiome profiles are minimally affected by collection method or DNA extraction protocols. Scientific Reports, 7, 8523. 28819242 10.1038/s41598-017-07885-3 PMC5561025

[cre2905-bib-0027] Maghini, D. G. , Moss, E. L. , Vance, S. E. , & Bhatt, A. S. (2021). Improved high‐molecular‐weight DNA extraction, nanopore sequencing and metagenomic assembly from the human gut microbiome. Nature Protocols, 16, 458–471. 33277629 10.1038/s41596-020-00424-x PMC8750633

[cre2905-bib-0028] Marotz, C. A. , Sanders, J. G. , Zuniga, C. , Zaramela, L. S. , Knight, R. , & Zengler, K. (2018). Improving saliva shotgun metagenomics by chemical host DNA depletion. Microbiome, 6, 42. 29482639 10.1186/s40168-018-0426-3 PMC5827986

[cre2905-bib-0029] McMurdie, P. J. , & Holmes, S. (2013). phyloseq: An R package for reproducible interactive analysis and graphics of microbiome census data. PLoS One, 8, e61217. 23630581 10.1371/journal.pone.0061217 PMC3632530

[cre2905-bib-0030] Moss, E. L. , Maghini, D. G. , & Bhatt, A. S. (2020). Complete, closed bacterial genomes from microbiomes using nanopore sequencing. Nature Biotechnology, 38, 701–707. 10.1038/s41587-020-0422-6 PMC7283042 32042169

[cre2905-bib-0031] Na, H. S. , Song, Y. , Yu, Y. , & Chung, J. (2023). Comparative analysis of primers used for 16S rRNA gene sequencing in oral microbiome studies. Methods and Protocols, 6, 71. 37623922 10.3390/mps6040071 PMC10460062

[cre2905-bib-0032] Okahashi, N. , Nakata, M. , Kuwata, H. , & Kawabata, S. (2022). Oral mitis group streptococci: A silent majority in our oral cavity. Microbiology and Immunology, 66, 539–551. 36114681 10.1111/1348-0421.13028

[cre2905-bib-0033] Paulson, J. N. , Stine, O. C. , Bravo, H. C. , & Pop, M. (2013). Differential abundance analysis for microbial marker‐gene surveys. Nature Methods, 10, 1200–1202. 24076764 10.1038/nmeth.2658 PMC4010126

[cre2905-bib-0034] Peng, X. , Cheng, L. , You, Y. , Tang, C. , Ren, B. , Li, Y. , Xu, X. , & Zhou, X. (2022). Oral microbiota in human systematic diseases. International Journal of Oral Science, 14, 14. 35236828 10.1038/s41368-022-00163-7 PMC8891310

[cre2905-bib-0035] Reynolds, L. J. , Roberts, A. P. , & Anjum, M. F. (2016). Efflux in the oral metagenome: The discovery of a novel tetracycline and tigecycline ABC transporter. Frontiers in Microbiology, 7, 220073. 10.3389/fmicb.2016.01923 PMC5138185 27999567

[cre2905-bib-0036] Roberts, A. P. , & Kreth, J. (2014). The impact of horizontal gene transfer on the adaptive ability of the human oral microbiome. Frontiers in Cellular and Infection Microbiology, 4, 124. 25250243 10.3389/fcimb.2014.00124 PMC4157583

[cre2905-bib-0037] Roberts, A. P. , & Mullany, P. (2010). Oral biofilms: A reservoir of transferable, bacterial, antimicrobial resistance. Expert Review of Anti‐Infective Therapy, 8, 1441–1450. 21133668 10.1586/eri.10.106

[cre2905-bib-0038] Saladié, M. , Caparrós‐Martín, J. A. , Agudelo‐Romero, P. , Wark, P. A. B. , Stick, S. M. , & O'Gara, F. (2020). Microbiomic analysis on low abundant respiratory biomass samples; improved recovery of microbial DNA from bronchoalveolar lavage fluid. Frontiers in Microbiology, 11, 572504. 33123104 10.3389/fmicb.2020.572504 PMC7573210

[cre2905-bib-0039] Salazar, O. , & Asenjo, J. A. (2007). Enzymatic lysis of microbial cells. Biotechnology Letters, 29, 985–994. 17464453 10.1007/s10529-007-9345-2

[cre2905-bib-0040] Sedghi, L. , DiMassa, V. , Harrington, A. , Lynch, S. V. , & Kapila, Y. L. (2021). The oral microbiome: Role of key organisms and complex networks in oral health and disease. Periodontology 2000, 87, 107–131. 34463991 10.1111/prd.12393 PMC8457218

[cre2905-bib-0041] Sohrabi, M. , Nair, R. G. , Samaranayake, L. P. , Zhang, L. , Zulfiker, A. H. M. , Ahmetagic, A. , Good, D. , & Wei, M. Q. (2016). The yield and quality of cellular and bacterial DNA extracts from human oral rinse samples are variably affected by the cell lysis methodology. Journal of Microbiological Methods, 122, 64–72. 26812577 10.1016/j.mimet.2016.01.013

[cre2905-bib-0042] Stinson, L. F. , Keelan, J. A. , & Payne, M. S. (2018). Comparison of meconium DNA extraction methods for use in microbiome studies. Frontiers in Microbiology, 9, 306524. 10.3389/fmicb.2018.00270 PMC5826226 29515550

[cre2905-bib-0043] Sui, H. , Weil, A. A. , Nuwagira, E. , Qadri, F. , Ryan, E. T. , Mezzari, M. P. , Phipatanakul, W. , & Lai, P. S. (2020). Impact of DNA extraction method on variation in human and built environment microbial community and functional profiles assessed by shotgun metagenomics sequencing. Frontiers in Microbiology, 11, 953. 32528434 10.3389/fmicb.2020.00953 PMC7262970

[cre2905-bib-0044] Sukumar, S. , Wang, F. , Simpson, C. A. , Willet, C. E. , Chew, T. , Hughes, T. E. , Bockmann, M. R. , Sadsad, R. , Martin, F. E. , Lydecker, H. W. , Browne, G. V. , Davis, K. M. , Bui, M. , Martinez, E. , & Adler, C. J. (2023). Development of the oral resistome during the first decade of life. Nature Communications, 14, 1291. 10.1038/s41467-023-36781-w PMC9998430 36894532

[cre2905-bib-0045] Tansirichaiya, S. , Mullany, P. , & Roberts, A. P. (2016). PCR‐based detection of composite transposons and translocatable units from oral metagenomic DNA. FEMS Microbiology Letters, 363, fnw195. 27521260 10.1093/femsle/fnw195 PMC5024762

[cre2905-bib-0046] Tansirichaiya, S. , Mullany, P. , & Roberts, A. P. (2019). Promoter activity of ORF‐less gene cassettes isolated from the oral metagenome. Scientific Reports, 9, 8388. 31182805 10.1038/s41598-019-44640-2 PMC6557892

[cre2905-bib-0047] Tansirichaiya, S. , Reynolds, L. J. , Cristarella, G. , Wong, L. C. , Rosendahl, K. , & Roberts, A. P. (2017). Reduced susceptibility to antiseptics is conferred by heterologous housekeeping genes. Microbial Drug Resistance, 22, 105–112. 10.1089/mdr.2017.0105 28604259

[cre2905-bib-0048] Tansirichaiya, S. , Winje, E. , Wigand, J. , & Al‐Haroni, M. (2022). Inverse PCR‐based detection reveal novel mobile genetic elements and their associated genes in the human oral metagenome. BMC Oral Health, 22, 210. 35624467 10.1186/s12903-022-02209-y PMC9137128

[cre2905-bib-0049] Teng, F. , Darveekaran Nair, S. S. , Zhu, P. , Li, S. , Huang, S. , Li, X. , Xu, J. , & Yang, F. (2018). Impact of DNA extraction method and targeted 16S‐rRNA hypervariable region on oral microbiota profiling. Scientific Reports, 8, 16321. 30397210 10.1038/s41598-018-34294-x PMC6218491

[cre2905-bib-0050] Tighe, S. , Afshinnekoo, E. , Rock, T. M. , McGrath, K. , Alexander, N. , McIntyre, A. , Ahsanuddin, S. , Bezdan, D. , Green, S. J. , Joye, S. , Stewart Johnson, S. , Baldwin, D. A. , Bivens, N. , Ajami, N. , Carmical, J. R. , Herriott, I. C. , Colwell, R. , Donia, M. , Foox, J. , … Mason, C. E. (2017). Genomic methods and microbiological technologies for profiling novel and extreme environments for the extreme microbiome project (XMP). Journal of Biomolecular Techniques, 28, 31–39. 28337070 10.7171/jbt.17-2801-004 PMC5345951

[cre2905-bib-0051] Vartoukian, S. R. , Adamowska, A. , Lawlor, M. , Moazzez, R. , Dewhirst, F. E. , & Wade, W. G. (2016). In vitro cultivation of ‘unculturable’ oral bacteria, facilitated by community culture and media supplementation with siderophores. PLoS One, 11, e0146926. 26764907 10.1371/journal.pone.0146926 PMC4713201

[cre2905-bib-0052] v. Wintzingerode, F. , Göbel, U. B. , & Stackebrandt, E. (1997). Determination of microbial diversity in environmental samples: Pitfalls of PCR‐based rRNA analysis. FEMS Microbiology Reviews, 21, 213–229. 9451814 10.1111/j.1574-6976.1997.tb00351.x

[cre2905-bib-0053] Wade, W. G. (2011). Has the use of molecular methods for the characterization of the human oral microbiome changed our understanding of the role of bacteria in the pathogenesis of periodontal disease? Journal of Clinical Periodontology, 38(Suppl. 11), 7–16. 21323699 10.1111/j.1600-051X.2010.01679.x

[cre2905-bib-0054] Wigand, J. , Tansirichaiya, S. , Winje, E. , & Al‐Haroni, M. (2021). Functional screening of a human saliva metagenomic DNA reveal novel resistance genes against sodium hypochlorite and chlorhexidine. BMC Oral Health, 21, 632. 34886820 10.1186/s12903-021-02000-5 PMC8656073

[cre2905-bib-0055] Wingett, S. W. , & Andrews, S. (2018). FastQ Screen: A tool for multi‐genome mapping and quality control. F1000Research, 7, 1338. 30254741 10.12688/f1000research.15931.1 PMC6124377

[cre2905-bib-0056] Zhang, L. , Chen, T. , Wang, Y. , Zhang, S. , Lv, Q. , Kong, D. , Jiang, H. , Zheng, Y. , Ren, Y. , Huang, W. , Liu, P. , & Jiang, Y. (2022). Comparison analysis of different DNA Extraction methods on suitability for long‐read metagenomic nanopore sequencing. Frontiers in Cellular and Infection Microbiology, 12, 919903. 35837476 10.3389/fcimb.2022.919903 PMC9273838

[cre2905-bib-0057] Zhang, L. , Huang, W. , Zhang, S. , Li, Q. , Wang, Y. , Chen, T. , Jiang, H. , Kong, D. , Lv, Q. , Zheng, Y. , Ren, Y. , Liu, P. , Jiang, Y. , & Chen, Y. (2022). Rapid detection of bacterial pathogens and antimicrobial resistance genes in clinical urine samples with urinary tract infection by metagenomic nanopore sequencing. Frontiers in Microbiology, 13, 858777. 35655992 10.3389/fmicb.2022.858777 PMC9152355

